# Antimicrobial Activity and Chemical Composition of Essential Oils from Verbenaceae Species Growing in South America

**DOI:** 10.3390/molecules23030544

**Published:** 2018-03-01

**Authors:** Cristina M. Pérez Zamora, Carola A. Torres, María B. Nuñez

**Affiliations:** 1 National Council for Scientific and Technical Research (CONICET), Godoy Cruz 2290, Buenos Aires C1425FQB, Argentina; cristinaperez@uncaus.edu.ar (C.M.P.Z.); carito@uncaus.edu.ar (C.A.T.); 2 Pharmaceutical Technology Laboratory, Department of Basic and Applied Science, National University of Chaco Austral, Comandante Fernández 755, Presidencia Roque Sáenz Peña, Chaco 3700, Argentina; 3 Laboratory of Microbiology, Department of Basic and Applied Science, National University of Chaco Austral, Comandante Fernández 755, Presidencia Roque Sáenz Peña, Chaco 3700, Argentina

**Keywords:** aromatic plants, volatile constituents, chemotypes, antibacterial synergism

## Abstract

The Verbenaceae family includes 2600 species grouped into 100 genera with a pantropical distribution. Many of them are important elements of the floras of warm-temperature and tropical regions of America. This family is known in folk medicine, and its species are used as digestive, carminative, antipyretic, antitussive, antiseptic, and healing agents. This review aims to collect information about the essential oils from the most reported species of the Verbenaceae family growing in South America, focusing on their chemical composition, antimicrobial activity, and synergism with commercial antimicrobials. The information gathered comprises the last twenty years of research within the South American region and is summarized taking into consideration the most representative species in terms of their essential oils. These species belong to *Aloysia*, *Lantana*, *Lippia*, *Phyla*, and *Stachytarpheta* genera, and the main essential oils they contain are monoterpenes and sesquiterpenes, such as β-caryophyllene, thymol, citral, 1,8-cineole, carvone, and limonene. These compounds have been found to possess antimicrobial activities. The synergism of these essential oils with antibiotics is being studied by several research groups. It constitutes a resource of interest for the potential use of combinations of essential oils and antibiotics in infection treatments.

## 1. Introduction

The Verbenaceae family includes 2600 species grouped into 100 genera with pantropical distribution. The most significant number of species is found in Latin America where they occur in a wide array of ecosystems. This family involves herbs, shrubs, and a few trees. They are an important element in the flora of South America [1].

The distribution of the Verbenaceae family in America is varied: for example, the *Verbena* genus contains 250 species, and the majority is native to the Americas and Asia, *Glandularia* J.F. Gmel. is a genus distributed from North to South in America [2], and *Lantana* L. has spanned from the tropics to the subtropics of America and Africa [3], as also *Lippia* L. and *Priva Adans* [2]. Other genera seem to be distributed along warm-temperature and tropical regions of America, for instance, *Tamonea* Aubl., from Mexico and the Caribbean to northern South America, Brazil, and eastern Bolivia. Some genera are confined to the southern part of South America, i.e., *Urbania* Phil., *Acantholippia* Griseb., *Diostea* Miers, *Lampaya* Philippi ex Murillo genera, all of which are restricted to Argentina and Chile [2].

The plants of this family are known as aromatic species, for ornamental use, or in folk medicine since ancient times. Most of their properties are due to the essential oil (EO) produced by their secondary metabolism. The composition of EOs is highly variable and determines their physical, chemical, and biological properties together with their organoleptic characteristics, which therefore determine their commercial use [4].

Many EOs from Verbenaceae were studied by means of in vitro tests and demonstrated inhibition of bacteria, fungi, and yeasts. Many microorganisms related to human diseases show resistance to antibiotics because of the inappropriate use of these antimicrobials. Consequently, there is an urgent need to discover new active substances [5].

In the era of antibiotic resistance, the awareness of the importance of finding antimicrobial substances continues to grow. Over the past few decades, there has been a tendency to search for these components in natural sources, including plants, because the demand for natural products as an alternative to the conventional treatments has increased. Many representatives of the Verbenaceae family have medicinal uses closely related to bacterial infections, as they have antiseptic properties and can be used for the treatment of fever, wounds, diarrhea, bronchitis, sinusitis, tetanus. In addition, the main chemical compounds present in these plants are recognized for their antimicrobial action. Hence, the aim of this review was to gather information about the EOs obtained from species of the Verbenaceae family growing in South America, their chemical composition, and their antimicrobial effects. Many reports about ethnobotany of these species are shown in Table 1. This family includes several species with pharmacological and ornamental uses, especially those of the genera *Aloysia*, *Lantana*, *Lippia*, and *Stachytarpheta*. This family is known in folk medicine, and the main uses are as digestive, carminative, antipyretic, antitussive, antiseptic, and healing agents.

Regarding the antimicrobial activity against human pathogens carried out by EOs of species belonging to the Verbenaceae family in South America, the main genera studied were *Lippia* (51.79%), *Lantana* (26.79%) and *Aloysia* (16.03%). There are few works about *Stachytarpheta* (3.6%) and *Phyla* (1.8%). The main contributions were given by Brazil (86.11%) in most species of all genera of the family. Other contributions were supplied by Argentina (5.56%), Colombia (5.56%), and Peru (2.78%).

*Lippia alba* (34.48%) and *Lippia origanoides* (20.69%) were the most studied species of the *Lippia* genus, whereas *Lantana camara* (53.33%) and *Lantana montevidensis* (26.67%) were the most studied of the *Lantana* genus. Concerning the *Aloysia* genus, the most studied species were *Aloysia triphylla* (33.33%), *Aloysia polystachya* (22.22%), and *Aloysia gratissima* (22.22%).

## 2. Essential Oils from the Verbenaceae Family

Essential oils are heterogeneous mixtures that may contain many compounds at different concentrations. Each EO is characterized by some major compounds, which can reach high levels compared to other compounds present in trace amounts.

It is known that the occurrence of secondary metabolites with similar biological activities can be expected in phylogenetically related plants, which may contribute to the implementation of more rational approaches for the search of new substances with potential economic interest [54]. This section provides information on the chemical composition of five genera and several species of different genres of the Verbenaceae family.

The *Lantana* species presented β-caryophyllene in high concentration, with cubebene, elixene, and phellandrene as minor compounds [59]. The β-caryophyllene is a chemical marker for species belonging to the *Lantana* genus [60]. *Lantana camara* is the most widespread species of this genus. The chemical composition of *L. camara* EO plays a role in its biological activity; the β-caryophyllene and (*E*)-nerolidol chemotypes showed antimicrobial and cytotoxic activities [61].

The *Lippia* species, which are morphologically similar to the *Lantana* genus, contain limonene, citral, carvacrol, β-myrcene, camphor, and thymol as the main chemical components [59]. Some examples are *L. alba* EO composed of carvone and limonene (group I), citral and β-caryophyllene, found in *L. alba*, *L. citriodora*, and *L. dulcis* EOs (group II), and carvacrol, thymol, and *p*-cymene present in *L. origanoides* and *L. micromera* species (group III). The group IV is characterized by a greater presence of *p*-cymene and a small amount of carvacrol and thymol, as in *L. origanoides* EO [62]. The chemical composition of *L. alba* EO is an example of the variations in EOs’ components, which determines the existence of a large number of chemotypes. In different regions of Colombia, the citral, carvone, and limonene chemotypes were found while, in Uruguay, the linalool chemotype was identified [63]. *L. alba* species in Argentina present the linalool chemotype (up to 91%), citral chemotype (up to 76% with two subtypes, i.e., myrcene or limonene), piperitone chemotype (up to 37%), lippione chemotype (up to 50%), and dihydrocarvone chemotype [64]. Another example of the variable chemical composition of Verbenaceae EOs is *Lippia javanica*, which presents five chemotypes, namely, the myrcenone rich-type (36–62%), carvone rich-type (61–73%), piperitenone rich-type (32–48%), ipsenone rich-type (42–61%), and linalool rich-type (>65%) [65]. The EO of *L. graveolens* consists mainly of thymol (31.6%) and sesquiterpenes such as caryophyllene (4.6%) and caryophyllene oxide (4.8%) [66]. The EO from *L. origanoides* also shows diversity in its chemical composition. Several authors revealed the different chemotypes found in various South American countries. Vega-Vela et al. [67] described different chemotypes in several Colombian regions: chemotype B (rich in carvacrol), chemotypes C and D (rich in thymol), chemotype E (eucalyptol and α-phellandrene), chemotype F (*p*-cymene, eucalyptol or β-phellandrene trace, and thymol methyl ether), and chemotype G (thymol methyl ether, *p*-cymene, thymol, and γ-terpinene). On the other hand, Stashenko et al. [68] classified *L. origanoides* EO into three chemotypes according to their major components; they were: chemotype A (α and β-phellandrene, *p*-cymene, and limonene), chemotype B (carvacrol), and chemotype C (thymol). The comparison of these results allowed concluding that the chemical composition of the EOs obtained from different regions shows significant variation based on its main constituents [67,69]. This chemical diversity is related to monoterpene and sesquiterpene hydrocarbons. The principal compounds are aromatic monoterpenes such as thymol and carvacrol, and monoterpene hydrocarbons and their oxygenated compounds type thujene, pinene, and carene, which are present in a lower proportion. The sesquiterpenes and their oxygenated derivates (β-caryophyllene, humulene and germacrene) constitute about 15% of EOs [67].

The compositions of the EO of the aerial parts of some species of the *Aloysia* genus presented oxygenated sesquiterpenes as the main components (between 40% and 50%) followed by sesquiterpenic molecules (30–34%) [70]. The EO from the leaves of *A. gratissima* species, examined in Brazil, Uruguay, and Argentina, contained limonene, sabinene, α-pinene, β-bisabolene, and copaenol; the EO from the flowers presented a high percentage of pulegone (65.8%) and other featured components, such as limonene, spathulenol, α and β-thujene, dihydrocarvone, and menthone [71].

The compounds most found in *Phyla nodiflora* EO were mainly monoterpenoids (86%), with limonene and carvone as main components, while 3-methyltridecane represented the compound with the lowest concentration [72,73]. The volatile oil isolated from *Phyla dulcis* by steam distillation consisted of 19.2% hernandulcin, but no camphor was found in plants grown in Brazil, even when extracted by supercritical CO_2_ [74]. The *Phyla dulcis* EO from Brazil was high in β-caryophyllene (10.6%), 6-methyl-5-hepten-2-one (10.5%), hernandulcin (8.8%), α-copaene (8.6%), and δ-cadinene (7.2%). In addition, it contained β-cedrene and α-calacorene, compounds found in cedarwood oils [75].

There are very few data related to the chemical composition of the EOs of species belonging to the genus *Stachytarpheta*. The EO of *Stachytarpheta gesnerioides* is mainly composed of guaiol (53.5%), α-pinene (16.1%), and isocaryophyllene (1.7%) [76]. In the composition of the EO from the leaves of *Stachytarpheta mutabilis*, oxyquinone sesquiterpenes, such as those of the eudesman type, are present in a greater percentage [77]. 

## 3. Antimicrobial Activity

Aromatic plants have great importance for the pharmaceutical industry, although synthetic products replaced many of them. Plant species show inhibition of bacteria, fungi, and yeasts. Nowadays, the emerging problem of antibiotic resistance is a driving force behind multiple studies on the potential antibacterial activity of EOs and the development of novel preventive or therapeutic strategies for health.

Many aromatic plants are practically immune to the attack of herbivores because of the presence of bioactive metabolites. This fact makes them attractive for study in search of new antimicrobials. The active compounds may be present in the stems, leaves, roots, flowers, or fruits. Regarding the study of the Verbenaceae family, most of the works involved the leaves as a source of EO, but some studies also used flowers.

The diversity of techniques used makes it difficult to compare results from different groups of researchers. Several are the factors that influence the data found in published works related to this topic, namely, microbiological methods, techniques of EO extraction, plant part involved in the extraction, harvesting season, climatic and environmental conditions where the plants were cultivated, and others. Consequently, the differences observed between articles could not possibly reflect a real difference between EOs’ features. It has been found that there is no consensus about both the methodology for the evaluation of the antimicrobial activity of a natural product, and an acceptable value for the minimal inhibitory concentration (MIC). Deep discrepancies among researchers have been found when considering antibiotic-like concentrations or good antimicrobial potential, even when the EO activity consists in a weak inhibition. We consider that a comparison between the MIC values of EOs and commercial antibiotics is not applicable, because the former contain a mixture of compounds which can interact among them, whereas the latter are pure compounds. Aligiannis et al. [78], for example, classified the antibacterial activity of plant extracts on the basis of MIC results, indicating as strong inhibition that corresponding to MIC up to 500 μg/mL, moderate inhibition that characterized by MIC between 600 μg/mL and 1500 μg/mL, and weak inhibition that observed with MIC above 1600 μg/mL. In contrast, Holetz et al. [79] argued that MIC values lower than 0.1 mg/mL represent strong antimicrobial action, values between 0.1 mg/mL and 0.5 mg/mL indicate moderate antimicrobial activity, values between 0.5 and 1.0 mg/mL indicate weak action, while values above 1.0 mg/mL indicate inactive products. The analysis of the MIC values found in the studied EOs allows us to suggest that values less than or equal to 0.5 mg/mL represent strong activity, while those between 0.5 mg/mL and 1.0 mg/mL indicate moderate action. The values between 1.0 mg/mL and 2.0 mg/mL indicate weak action and those greater than 2.0 mg/mL should be considered as corresponding to lack of activity. This appreciation can be useful, since the previous work focused mainly on plant extracts and not on essential oils. Table 2 presents studies on the antimicrobial activity of EOs from the Verbenaceae family against human pathogens, carried out in South America in the last two decades. It should be noted that the broth microdilution test is the most used method to determine the MIC. These data are detailed in the following paragraphs. 

According to Sartoratto et al. [80], the EO from *A. triphylla* showed inhibition values between 0.05 and >2 mg/mL against Gram-positive bacteria but it was not active against Gram-negative bacteria. However, Duarte et al. [81] reported that *A. triphylla* inhibited 12 *Escherichia coli* serotypes, with MIC values between 400–1000 μg/mL, showing moderate activity. *A. polystachya* showed inhibition values between 3.64 and 29.13 μg/mL against Gram-positive and Gram-negative bacteria, but it was not active against *Pseudomonas aeruginosa* [82]. *A. gratissima* showed inhibition values between 1000 and 4000 μg/mL against Gram-positive and 2000–4000 μg/mL against Gram-negative bacteria. The minimal bactericidal concentration (MBC) was twice the MIC in most of the cases and was the same as the MIC only for *E. coli* [8]. *Aloysia sellowii* showed inhibition values between 1.7 and 16 mg/mL against Gram-positive bacteria and between 6.7 and >20 mg/mL for Gram-negative bacteria. The MBC values were between two and three times the MIC. *Aloysia sellowii* was active against yeasts with MIC values between 4–16 mg/mL and Minimal Letal Concentration between 4–>20 mg/mL [14]. 

*Lantana caatingensis* showed inhibition values between 64 and over 1024 μg/mL against Gram-positive and 256–512 μg/mL against Gram-negative bacteria [16]. The species *Lantana camara* showed inhibition values between 64 and 256 mg/mL against Gram-positive and Gram-negative bacteria [26], and above 1250 μg/mL against *Mycobacterium spp* [83]. *Lantana montevidensis* showed inhibition at 256 μg/mL against *Staphylococcus aureus* and at 512 μg/mL against *E. coli* [27]. 

The EO of *Lippia alba* was active against *Candida albicans* [84], six *E. coli* serotypes [81], pathogenic bacteria which contaminate food [85], filamentous fungi [86], and oral pathogens with MIC and MBC values between 0.006–3.2 mg/mL [31].

The EO of *Lippia sidoides* (EOLS) showed MIC of 128 μg/mL for *Staphylococcus aureus*, 256 μg/mL for *Streptococcus mutans*, *Klebsiella pneumoniae*, *Providencia rettigeri*, and *Enterobacter cloacae*, and 512 μg/mL for *Enterococcus faecalis*, *P. aeruginosa* and *E. coli* [87]. Also, EOLS was active against strains of *Candida albicans*, *Candida tropicalis*, *Candida* spp. These strains were isolated from dogs and cats. *Candida parapsilosis* (ATCC 22019) and *Candida krusei* (ATCC 6528) were also used in this assay. The MICs for *Candida* spp. strains ranged from 620 to 2500 mg/mL, and the MFCs ranged from 1250 to 5000 mg/mL [88]. Likewise, EOLS showed MIC values between 5 and 10 mg/mL and MBC values between 20–40 mg/mL against oral pathogens [50].

Other species mentioned in Table 2 were studied by means of the disk diffusion assay. Disks of 6 mm of diameter were impregnated with 10 or 15 μL of EO, resulting in inhibition halos between 8 and 20 mm diameter. Aguiar et al. [89] considered that halos with a 10 mm diameter represent a good inhibition. According to the observed results in the literature, halos with diameters larger than 20 mm are rare. However, there are reports with these values for *L. origanoides* [90,91], *Lippia gracilis* [92] and *Lippia grandis* [43]. The target bacteria generally used in this type of studies were *S. aureus*, *S. epidermidis*, *Bacillus cereus*, and *E. faecalis* (Gram-positives), *E. coli*, *P. aeruginosa*, *K. pneumoniae*, and *Salmonella* (Gram-negatives). The use of ATCC strains facilitates the comparison of the antibacterial potency between plants of the same species that grow in different regions. However, the use of clinical isolates gives a valuable contribution in this field, since it gives a more realistic scenario of the activity of EOs against pathogen strains.

## 4. Antimicrobial Synergism

Over the last decades, interactions between natural products and commercial antibiotics have been comprehensively studied. Many researchers have demonstrated the ability of natural products to regulate antibiotic activity possibly exerting a synergistic effect.

The checkerboard test evaluates the effect of interactions between two antimicrobial substances. This assay is one of the most commonly used to determine synergism. The MIC values of combinations are registered at one time point [106], and the fractional inhibitory concentration (FIC) index for the two antimicrobial substances are calculated. Sometimes, the results of this assay are interpreted by plotting an isobologram. Another test used to determine synergism is the time-kill assay. It involves measuring the number of viable bacteria in a liquid medium in the presence of a particular combination of antimicrobial substances at different time points. Although time-kill curves are not widely used to study antibacterial interactions, they can be considered a clinically relevant model if the concentrations used represent those achieved at the site of an infection [107]. As previously mentioned, for the determination of antibacterial activity, methods used to evaluate interactions between EOs and antibiotics differ widely, and this makes data comparison difficult. However, the use of FIC indexes allows comparisons of the results. The development of a more standardized method of serial passaging in sublethal concentrations of EO would enable a better investigation of the possible loss of sensitivity or cross-resistance [108].

The commercial antibiotics and bacterial strains most frequently used to evaluate a synergistic action are shown in Table 3. The most relevant results are described below.

Many examples of synergism can be found in the literature regarding *Lantana* species. The concentration of neomycin decreased 50% against multiresistant *E. coli* when this antimicrobial was combined with *L. caatingensis* EO. Similarly, there was a 75% decrease in the concentration of amikacin against *S. aureus* ATCC 12692 when this EO was present [16]. *L. camara* EO at 50 μg/mL increased the activity of amikacin up to 65% against *P. aeruginosa* and up to 29% against *S. aureus*. When this EO was combined with gentamicin, the antibiotic efficacy increased to 21% against *P. aeruginosa* and at none effect was detect against *S. aureus* [24]. *Lantana montevidensis* EO at 50 μg/mL improved the activity of gentamicin by 12% against *P. aeruginosa* ATCC 15442 and by 10% against *S. aureus* ATCC 12692. In addition, when this EO was combined with amikacin, the antibiotic efficacy increased up to 102% against *P. aeruginosa* ATCC 15442 and to 29% against *S. aureus* ATCC 12692 [25]. The combination of neomycin with *L. montevidensis* and *L. camara* EOs showed no interaction of the two components against *S. aureus* ATCC 6538 [26].

Likewise, *Lippia* species exerted several synergistic effects. The presence of 12% *L. alba* EO increased between 12.5% and 35.7% the activity of erythromycin against two *S. aureus* ATCC [37]. *Lippia gracilis* showed a modulatory effect on aminoglycoside activity. A reduction of the MIC value of gentamicin and amikacin against two *E. coli* strains and *S. aureus* were observed [40]. Moreover, the addition of 128 μg/mL of *L. origanoides* EO to the growth medium did cause a 10-fold decrease in the MIC of neomycin (2500–248 μg/mL) and amikacin (788–78 μg/mL). This demonstrated a synergistic effect between *L. origanoides* EO and aminoglycosides against the methicillin resistant *Staphylococcus aureus* (MRSA) strain [46].

The presence of *L. sidoides* EO at 50%, 25%, 12% and 6%, produced an increase of 429.41%, 349%, 256.82% and 21.53% in gentamicin activity against a *S. aureus* strain. The activity of amikacin and neomycin was enhanced when either antibiotic were combined with this EO [52]. Veras et al. [87] combined *L. sidoides* EO with aminoglycosides (gentamicin and neomycin) and β-lactams (penicillin G and ceftriaxone) and demonstrated indifferent and synergistic effects depending on the bacteria tested. The combination of the EO with aminoglycosides was synergistic on *S. aureus* and *P. aeruginosa* (the MIC was reduced four times)*.* Synergism was also detected when EO and gentamicin were used against *K. pneumoniae* (the MIC decreased from 32 to 1 μg/mL). This mix showed no effects on the other bacteria. Regarding the interactions between this EO and β-lactams, synergism was detected against *S. mutans* (EO and penicillin G) and *E. faecalis* (EO and ceftriaxone), whereas no effect was found against other bacteria. In the first case, there was a four-fold reduction in the MIC value; the MIC value decreased sixteen times when the EO was combined with ceftriaxone. Antagonistic interactions have not been reported in any of the papers analyzed in this work.

This body of evidence proves that plants of this family not only possess antibacterial activities but also can enhance the effects of antibiotics. The authors relate the potential antibiotic effect of EOs to the presence of monoterpenes and sesquiterpenes.

## 5. Relationship between the Chemical Composition of EOs and Their Antimicrobial Activity

The antimicrobial activity of EOs depends on their chemical composition and on the amount of the single components. Most EOs have a greater effect on Gram-positive bacteria than on Gram-negative bacteria. This behavior is attributed to the differences in the bacterial cell membrane composition. In Gram-positive bacteria, hydrophobic components easily penetrate the cell wall and act upon it as within the cytoplasm. Gram-negative bacteria have a peptidoglycan layer that is 2–3 nm thick, and an outer membrane (OM) lies outside the thin peptidoglycan layer and is firmly linked to it by Braun’s lipoprotein embedded in the OM. This is composed of a double layer of phospholipids that is linked to the inner membrane by lipopolysaccharides (LPS). The peptidoglycan layer is covered by an OM that contains various proteins as well as LPS, which makes the bacteria more resistant to EOs and other natural extracts with antimicrobial activity [107,109].

The mechanism of action of EOs depends on their chemical composition, and their antimicrobial activity is not attributable to a single mechanism; this is widely described by Nazzaro et al. in their work [109]. The effect of the chemical constituents depends on their amount in the EOs, i.e., at low concentrations, they can interfere with enzymes involved in the production of energy and, at higher concentrations, they can denature proteins [109,110]. Examples of chemical components of EO tested experimentally are discussed below.

As previously described, β-caryophyllene, citral, 1,8-cineole, linalool, thymol, limonene, and carvone are volatile substances present in several EOs extracted from plants with recognized antimicrobial properties. These compounds presented antimicrobial activity. The possible mechanisms of action of these compounds are described in the following paragraphs.

Citral exhibited antimicrobial activity against pathogenic and food-spoilage bacteria such as *E. coli* O157:H7, *Salmonella enterica* serovar Typhimurium, *Listeria monocytogenes*, and *Staphylococcus aureus* [111,112]. This compund disrupts and penetrates the lipid structure of the cell wall of bacteria. It leads to protein denaturation and destruction of the cell membrane, followed by cytoplasmic leakage, cell lysis and death [113]. Citral was reported in the EO of *L. alba* (citral chemotype).

Linalool is one of the main components of some of these EOs (*A. sellowii*, *L. alba*) and it was previously reported to cause an increased permeability not only of the negatively charged membranes but also of fungal cells [114,115]. Because of the nature of their chemical structure, alcohols possess a strong binding affinity to different molecular structures, such as proteins or glycoproteins. Hence, they have great affinities for cell membranes and exhibit high potential to permeate cell walls, leading to the leakage of cytoplasmic material [116,117].

Thymol is one of the monoterpene phenols present in EOs from plants belonging to the Verbenaceae family (*A. triphylla*, *L. graciliis*, *L. grandis*, *L. origanoides*, *L. sidoides*). Its biological activities include antioxidant, anti-inflammatory, local anaesthetic, antinociceptive, cicatrizing, antiseptic activity, and especially antibacterial and antifungal properties [118]. Some authors [118,119] speculated that the antimicrobial effect of thymol might result, at least in part, from a perturbation of the lipid fraction of the bacterial plasma membrane, resulting in the leakage of intracellular materials. Xu et al. [120] confirmed it, because they demonstrated that this natural compound induces the permeabilization and depolarization of the cytoplasmic membrane. In addition, Chauhan & Kang [121] evaluated the antimicrobial properties and mechanism of action of thymol against *S. typhimurium* and showed the disruption of membrane integrity. They concluded that this is the main mechanism of action of thymol.

Germacrene-D is an organic compound belonging to the class sesquiterpenoid germacrane. The sesquiterpene hydrocarbon germacrene has five isomers, i.e., Germacrene A, B, C, D, and E. Germacrene D possesses antibacterial properties [122,123]. This sesquiterpene is present in several of the EOs studied, among which we can mention EOs of *A. gratissima*, *L. camara*, *L. montevidensis*, *L. alba*. 

The EO of *L. alba* and its main components, such as citral and carvone, presented antibacterial and antibiofilm activities against *S. aureus*. The lowest MIC and MBC values were 0.5 mg/mL when *L. alba* EOs, citral, and carvone were used. The inhibition (100%) of *S. aureus* biofilm formation and the elimination of biofilm cells were confirmed. No elimination of biofilm cells was observed when carvone was used. Carvone is a monoterpene reported as one of the most effective antimicrobial agents present in several plants. Its main mechanism of action comprises the destabilization of the structure of phospholipids and the interaction with membrane proteins, and it acts as a proton exchanger reducing the pH gradient across the membrane [124].

## 6. Conclusions

Essential oils of South American plants from the Verbenaceae family contain as their main components monoterpenes and sesquiterpenes, such as thymol, β-caryophyllene, citral, 1,8-cineole, carvone, and limonene. The presence of these compounds, which increase or alter the permeability of bacterial membranes, could explain their antimicrobial action and their synergistic effect with antibiotics.

Pharmaceutical industries are in need of eco-friendly alternatives to drug molecules to treat infectious diseases. Thus, these EOs might be a prospective source of alternative antimicrobial agents and may play an important role in the discovery of new drugs against a wide range of pathogenic microorganisms in the near future.

## Figures and Tables

**Table 1:** Ethnobotany of Verbenaceae family plants.

Scientific name	Vernacularar Name	Country	Used Part	Popular Use	Reference
*Acantholippia seriphioides* (A. Gray) Moldenke	Tomillo de campo	Argentina	Not specified	Digestive, antipyretic, and to treat cold.	[6]
*Aloysia castellanosii* Moldenke	No data	Argentina	Leaves Herbal tea	Digestive and antispasmodic.	[7]
*Aloysia catamarcensis* Moldenke	No data	Argentina	Leaves Herbal tea	Digestive and antispasmodic.	[7]
*Aloysia gratissima* (Gillies & Hook.) Tronc. var. chacoensis (Moldenke) Botta	Erva santa, poleo del campo.	Brazil, Argentina	Leaves	To treat symptoms associated with headaches, bronchitis, digestive disorders, and nervous disorders.	[8,9,10,11]
*Aloysia polystachya*	Poleo real, té de burro, burrito.	Argentina, Brazil	Leaves, flowery summits.	Digestive, carminative, hypertensive, sedative.	[12,13]
*Aloysia sellowii* (Briq.) Moldenke (Syn.: *Lippia affinis* Briq.)	Garupá, cidrozinho or erva de sepultura.	Brazil		Diuretic, digestive, to treat cold, influenza, and respiratory disorders.	[14]
*Aloysia triphylla* (L´Hér.) Britton	Cedrón	Argentina	Leaves	Digestive, carminative, and tonic.	[13]
*Aloysia virgata* (Ruiz & Pav.) *Pers. Aloysia virgata* var. platyphylla (Briquet) Mold.	Niño rupá guazú or pa’ira yvoty.	Argentina	Not specified	Not specified.	[15]
*Glandularia incisa* (Hook.) Tronc.	Margarita	Argentina	Leaves	Antipyretic	[14]
*Lantana caatingensis* Moldenke	No data	Brazil		Antirheumatic, antipyretic, to treat wounds and intestinal colics.	[16]
*Lantana camara* L. (Syn. *Lantana mutabilis* Weigel, *L. aculeata* L., *Camara aculeata* Kuntze, C. vulgaris Benth.)	Yerba de la maestranza, siete colores, lantana, camará, camara de spinho, camará-vermelho, camará-branco, camará-juba, camarazinho, cambará-cambará -de-cheiro, erva-chumbinho, erva-sagrada, capitão-do-campo, chá-de-pedestre.	Peru, Brazil.	Leaves, flowers, and roots.	To treat tetanus, malaria, tumors, rheumatism. Symptoms of itches, dermatitis, ulcers, swellings, catarrh, dysentery, eczema, fever, influenza, asthma, and roughor bronchitis. Emmenagogue, carminative, emetic, sudorific, diuretic, expectorant, febrifuge, anticonvulsivant, antiseptic.	[17,18,19,20,21,22,23,24]
*Lantana fucata* Lindl.	Cidreira brava	Brazil.	Leaves and flowers.	Tonic, digestive, for pruritus, ulcers, swellings, biliary fever, rheumatism, antiseptic for wounds.	[22]
*Lantana montevidensis* Briq.	Cambará	Brazil.	Leaves.	Digestive, biliary fever. Toothache, bronchitis, antiseptic for wounds, treatment of pruritus.	[25,26,27]
*Lippia alba* (Mill.) N.E. Br. ex Britton and P. Wilson. (Syn.: *Lantana alba* Mill., *Lantana geminata* (Kunth) Spreng., *Lippia geminata* Kunth, *Lippia geminata* var. microphylla Griseb., *Lippia globiflora* var. geminata (Kunth) Kuntze).	Lemon balm, bushy matgrass, bushy lippia, pitiona, erva-cidreira, cidrila, chá-de-tabuleiro, cidreira-brava, alecrim-selvagem, falsa-melissa, alecrim-do-campo, erva-cidreira-de-campo, salva-do-brasil, salva-limão, salvia de castilla, salvia morada, carmelita.	Brazil, Argentina.	Leaves.	Digestive, anti-spasmodic, emmenagogue. To treat diarrhea, cough, asthma, and fever. Antipyretic, analgesic, and sedative.	[13,28,29,30,31,32,33,34,35,36,37]
*Lippia brasiliensis* (Link) T. Silva (Syn.: *Camara brasiliensis* (Link) Kuntze; *Lantana brasiliensis* Link; *Lantana cinerea* Lam. ex Otto & Dietr; *Lantana longifolia* Mart. ex Colla; *Lantana pernambucensis* Moldenke; *Lantana spicata* Vell.	Yarabisco, sucupira, yerba sagrada.	Brazil, Peru, Paraguay and Venezuela.	Leaves and flowers.	No data.	[38]
*Lippia gracilis* Schauer (Syn: *Lippia af.f gracillis*)	Alecrim-da-chapada, alecrim-de-serrote, alecrim-de-tabuleiro.	Brazil	Leaves	To treat cough, bronchitis, nasal congestion, headache, flu, sinusitis, jaundice, and paralysis, antimicrobial and antiseptic.	[39,40,41,42]
*Lippia grandis* Schauer	Erva-do-marajó	Brazil	Leaves	Treatment of disorders of the liver and stomach.	[43]
*Lippia integrifolia*	Incayuyo	Argentina	Not specified	Dyspepsia, indigestion, stomachaches, diuretic, emmenagogue, tonic agent.	[44]
*Lippia microphylla* Cham. (Syn.: *L. microphylla* Cham. and Schlecht.; *L. microphylla* Mart.; *Lantana microphylla* Mart. ex Uphof.).	“alecrim-da- chapada,” “alecrim-de-tabuleiro,” “alecrim-pimenta,” and “alecrim-do-mato”.	Brazil	Leaves	To treat gastrointestinal disorders, influenza, bronchitis, cough, nasal congestion, and sinusitis.	[45]
*Lippia origanoides* H.B.K. (Syn.: *L. berterii* Spreng., *Lippia schomb urgkiana* Schauer)	Oregano de monte	Colombia and Brazil.	Not specified	Stomach pains, indigestion, heartburn, diarrhea, nausea, flatulence. To treat respiratory disease, menstrual cramps, Antiseptic for mouth, throat, and wound.	[46,47]
*Lippia sidoides* Cham. (Syn.: *L. multicapitata* Mart.)	“Alecrim-pimenta” and ”alecrim do campo”.	Brazil	Leaves	To treat acne, antiseptic on skin and mucosal tissues, inflammation of the gums. For rhinitis, influenza, colds, pulmonary.	[48,49,50,51,52,53,54]
*Lippia trifolia*		Brazil	Leaves	Treatment of respiratory disorders and as a sedative.	[54]
*Lippia turbinata* var. integrifolia Griseb. (Syn.: *L. integrifolia* (Griseb.) Hieron.)	“poleo”	Argentina	Leaves and flowery summits.	Digestive and antispasmodic. Dyspepsia, oliguria and dysmenorrhea.	[9,44]
*Phyla dulcis* (Syn.: *Lippia dulcis*)	Aztec sweet herb, honey herb and Mexican lippia.	Brazil	Herbal as infusion	Treatment of cough, colds, bronchitis, asthma. Antispasmodic activity.	[55]
*Phyla reptans* (Kunth) Greene	Mosko yuyo	Argentina	Leaves	To treat infected wounds, skin rashes.	[9]
*Stachytarpheta cayennensis* (Rich.) Vahl	Gerbão	Brazil	Leaves and flowers.	Purgative, vermifuge, expectorant, diuretic, emmenagogue, liver disorder anti-inflammatory, antihelmintic, and antiulcerogenic.	[56,57,58]

**Table 2:** Antimicrobial activity of essential oils (EOs) of plants belonging to the Verbenacea family against human pathogens (bacteria, fungi, or yeasts).

Botanical Name	Majority Compounds	Organisms inhibited			MIC Value/Range	Reference
		Gram Positive	Gram Negative	Fungy/Yeast		
*Aloysia gratissima*	No data.	-	-	*Candida albicans* ATCC 10231	>2 mg/mL ^▲^	[84]
	β-pinene, *E*-pinocamphone, Z-pinocamphone, *E*-pinocarveol acetate, α-caryophyllene, guaiol	*Streptococcus sanguis* ATCC 10556, *S. mitis* ATCC 903	*Fusobacterium nucleatum*, ATCC 25586, *Porphyromonas gingivalis* ATCC 33277	*C. albicans* CBS 562	0.015–0.5 mg/mL ^▲^	[93]
	1,8-cineole, germacrene-D, β-caryophyllene, and β-pinene	*S. aureus* ATCC 25923, *Bacillus cereus* ATCC 11778	*Acinetobacter baumanii* ATCC 17978, *E. coli* ATCC 25922, *P. aeruginosa* ATCC 27853	*Cryptococcus neoformans* ATCC 32264, *C. albicans* ATCC 10231, *Aspergillus flavus* ATCC 9170, *A. fumigates* ATCC 26934, *Rhizopus* sp. CL 35, *Microsporum canis* C112, *M. gypseum* C115, *Trichophyton mentagrophytes* ATCC 9972, *T. rubrum* C137, *Epidermophyton floccosum* C114	1000–4000 μg/mL ^▲^	[8]
*Aloysia polystachya*	carvone, limonene	*Staphylococcus aureus* ATCC 25923, *S. aureus* ATCC 29213, *S. epidermidis* ATCC 12228, *Enterococcus faecalis* ATCC 29212	*Escherichia coli* ATCC 35218, *Pseudomonas aeruginosa* ATCC 27853, *Klebsiella pneumoniae*, *Enterobacter cloacae*	-	3.64–29.13 μL/mL ^▲^	[82]
*Aloysia sellowii*	1,8-cineole, carvacrol, terpinen-4-ol, linalool, β-pinene, R (+)-limonene, myrcene	*S. aureus*, *S. epidermidis. Micrococcus luteus*	*K. pneumoniae*, *E. coli, Salmonella Setubal*	*Saccharomyces cerevisiae*, *C albicans*	1.7– >20 mg/mL ^▲^	[14]
*Aloysia triphylla*	oxygenated monoterpenes	*B. cereus*, *S. aureus* ATCC 25212, *S. epidermidis*, *M. luteus* ATCC 9341, *Enterococcus faecalis* ATCC 29212	*E. coli*, *P. aeruginosa*, *Klebsiella* sp., *Proteus mirabilis*	*C. albicans*	28.12–450 μg/disk ^♣^	[13]
	monoterpenes, thymol, geranial, neral, limonene	*Rhodococcus equi* CCT0541, *M. luteus* CCT2692, *S. aureus* CCT2740, *S. epidermidis* ATCC12228, *B. subtilis Cohn* CCT2576, *Enterococcus faecium* ATCC10541 Schleifer Kilpper-Balz (registered at ATCC as *Streptococcus faecium*), *Enterococcus faecium* CCT5079, clinical isolates of genito-urinary infections *S. aureus*, and *Enterococcus* sp.	*P. aeruginosa* ATCC13388, *Salmonella choleraesuis* CCT4296, *E. coli* CCT0547, clinical isolates of genito-urinary infections: *E. coli*, *Klebsiella ozaenae*, *Enterobacter aerogenes*, *Proteus mirabilis.*	*C. albicans* ATCC 10231	0.05– >2 mg/mL ^▲^	[80]
	No data	-	*E. coli* (13 serotypes clinical strains)	-	400–1000 μg/mL ^▲^	[81]
	*E*-pinocarveol, guaiol, bulnesol	*S. sanuis* ATCC 10556, *S. mitis* ATCC 903	*F. nucleatum*, ATCC 25586, *Porphyromonas gingivalis* ATCC 33277	*C. albicans* CBS 562	0.015–0.5 mg/mL ^▲^	[93]
	geranial, neral, limonene, camphor, cariophyllene oxide, spathulenol	*B. cereus*, *E. faecalis*, *M. luteus*, *S. aureus*, *S. epidermidis*	*E. coli*, *K. pneumoniae*, *P. mirabilis*, *P. aeruginosa*	*Rhodotorula* sp, *Hansenula* sp, *C. Albicans*	7–900 mg/mL ^♣^	[94]
	geranial, neral, geraniol, biciclogermacreno, nerol	*Staphylococcus aureus* and *Enterococcus* sp.	*Escherichia coli*, *Klebsiella ozaenae*, *Enterobacter aerogenes*, *Proteus mirabilis*	-	10–50 μg/mL ^♣^	[95]
	geranial, neral, limonene, caryophyllene oxide, spathulenol	*S. aureus* ATCC 25923	-	-	2.3–200 μg/mL ^▲^	[96]
*Aloysia virgata*	β-caryophyllene	*S. aureus* ATCC 25923, *Bacillus cereus* Ribotype 1 222-173-S4	*E. coli* ATCC 11229	-	0.125% (*v*/*v*) ^▲^	[54]
*Lantana caatingensis*	β-caryophyllene, spathulenol, and bicyclogermanecrene	*S. aureus* ATCC 6538, ATCC 12692, and 358 (multiresistant clinical strain)	*E. coli* ATCC 25922, and 27 (multiresistant clinical strain), *P. aeruginosa* ATCC 15442	-	64– ≥1024 μg/mL ^▲^	[16]
*Lantana camara*	carvone, limonene	*Listeria monocytogenes* CCMI 1106, *M. luteus* CCMI 322, *S. faecium* CCMI 338, *S. aureus* CCMI 335	*E. coli* CCMI 270, *P. aeruginosa* CCMI 331	-	≥200 μg/mL ^●^	[17]
	bicyclogermacrene, isocaryophyllene, valencene, germacrene-D	*S. aureus* ATCC 10390	*E. coli* ATCC 25922, *P. vulgaris* ATCC 13315, *P. aeruginosa* ATCC 15442, *Vibrio cholerae* ATCC 15748	-	0.62–10% (*v*/*v*) ^♣^	[20]
	germacrene-D	*S. aureus* ATCC 25923, *S. sanguinis* ATCC 49456	*E. coli* ATCC 25922, *S. tiphymurium* ATCC 14028	*C. albicans* ATCC 18804, *A. flavus* CCT 4952, *F. proliferatum* CML 3287	3.91–125 μg/mL ^♣^	[21]
	isocaryophyllene, valencene, germacrene-D	*S. aureus* ATCC 6538, *P. vulgaris* ATCC 13135	*Escherichia coli* ATCC 2992, *Pseudomonas aeruginosa* ATCC 5442, *V. cholerae* ATCC 15748	-	64– ≥512 μg/mL ^▲^	[26]
	germacrene-D, β-caryophyllene	*S. aureus* ATCC 25923, *B. cereus* Ribotype 1 222-173-S4	*E. coli* ATCC 11229	-	5 μL/disk ^♣^	[54]
	germacrene-D, β -caryophyllene, farnesene	*Mycobacterium* spp. (smegmatis, chelonae, tuberculosis)	-	-	>1250 μg/mL ^▲^	[84]
	bicyclogermacrene, β-caryophyllene	-	-	*Candida krusei* ATCC 6258, *C. albicans* (clinical isolates)	12.5 mg/mL ^♣^	[97]
	No data	*S. aureus*	*E. coli*	-	289.7 μg/mL ^◊^	[98]
*Lantana montevidensis*	β-caryophyllene, germacrene-D, bicyclogermacrene	*S. aureus* ATCC 6538	*E. coli* ATCC 2992, *P. aeruginosa* ATCC 5442, *P. vulgaris* ATCC 13135, *V. cholerae* ATCC 15748	-	128– ≥512 μg/mL ^▲^	[26]
	germacrene-D	*S. aureus* ATCC 25923, *B. cereus*, Ribotype 1 222-173-S4	*E. coli* ATCC 11229		5 μL/disk ^♣^	[54]
*Lippia alba*	limonene, carvone, geranial, neral	-	*Porphyromonas gingivalis*, *Aggregatibacter actinomycetemcomitans* ATCC 43717, *Fusobacterium nucleatum* ATCC 25586, *Bacterioides fragilis* ATCC 25285	-	0.00625– >3.2 mg/mL ^▲^	[31]
	No data	-	*E. coli* (13 serotypes clinical strains)	-	400– >1000 μg/mL ^▲^	[81]
	Linalool	-	-	*C. albicans* ATCC 10231	0.6 mg/mL ^▲^	[84]
	geranial, neral, d-limonene, germacrene-D, g-terpinene, α-pinene, citronellal, α-phellandrene, α-copaene	*S. aureus* ATCC 6538P, *L. monocytogenes* ATCC 33090, *L. innocua* ATCC 19115	*E. coli* ATCC 10536, *P. aeruginosa* ATCC 9027, *S. choleraesuis* ATCC 10708	-	0.29–9.37 mg/mL ^▲^	[85]
	Linalool	-	-	*Trichophytum ribrum*, *Microsporum gypseum*, *Epidermophytum floccosum*	39–312 μg/mL ^▲^	[86]
	No data	*S. sanguis* ATCC 10556, *S. mitis* ATCC 903	*F. nucleatum* ATCC 25586, *Porphyromonas gingivali*, ATCC 33277	*C. albicans* CBS 562	0.25– >1 mg/mL ^▲^	[93]
	carvone, piperitenone, piperitone, citral	*S. aureus*	*Escherichia coli*, *Salmonella tiphymurium*	-	No active	[99]
	Geraniol, geranial, neral	*S. mutans* ATCC 35668	-	-	0.001 mg/ 100 mL ^♠^	[100]
*Lippia brasiliensis*	hydrocarbons sesquiterpens: β-caryophyllene, germacrene-D, and bicyclogermacrene	*S. aureus* ATCC 25923, *Bacillus cereus* Ribotype 1 222-173-S4	*E. coli* ATCC 11229	-	0.125% (*v*/*v*) ^♣^	[54]
*Lippia gracillis*	thymol, methyl thymol, β-caryophyllene, carvacrol, p-cymene, γ-terpinene	-	-	*Tricophytum rubrum* H6 (ATCC-MYA3108), 2 clinical isolated *T. rubrum*	11.72–93.75 μg/mL ^▲^	[42]
	Carvacrol	*S. aureus*	*Morganella morganii, Salmonella sp*, *Salmonella paratiphy*, *Serratia mercescens*, *P. mirabilis*, *K. pneumoniae*, *E. coli*, *P. aeruginosa*	-	5 μg/mL ^♣^	[92]
*Lippia grandis*	carvacrol, p-cymene, thymol	*S. aureus* ATCC 25923, *Enterococcus faecalis* ATCC 51299, *E. faecalis* ATCC 29212	*E. coli* ATCC 35218, *E. coli* ATCC 25922, *K. pneumoniae* ATCC 700603, *P. aeruginosa* ATCC 27953	*C. albicans* (clinical isolated)	0.57–1.15 mg/mL ^▲^	[43]
*Lippia origanoides*	carvacrol, γ-terpinene, thymol, methyl thymol, p-cymene	*S. aureus* ATCC 25923, *S. aureus* MRSA	*E. coli* ATCC 25922	*C. albicans*, *C. tropicalis*	10 μL ^♣^	[90]
	Carvacrol	*S. mutans* ATCC 25175, *S. aureus* ATCC 25923, *S. aureus* MRSA (BMB9393), *Lactobacillus casei* ATTC 4646	-	*C. albicans* Serotype B ATCC 36802, *C. albicans*, *C. guilliermondii*, *C. parapsilosis*, *Cryptococcus neoformans* T1-444 Serotype A, *Trichophytum rubrum* T544, *Fonsecaea pedrosoi* 5VPL	10 μL *	[91]
	phenolic compounds: thymol and carvacrol	*S. aureus*	*E. coli*, *S. Tiphymurium*	-	No data ^♦^	[99]
	o-cymene thymol methyl ether carvacrol	*S. aureus* ATCC 25923	*E. coli* ATCC 25922, *S. cholerasuis* ATCC10708	-	30–120 μL/mL ^●^	[101]
	Carvacrol	*S. aureus* ATCC 6538	*E. coli* ATCC 6538, *P. aeruginosa* ATCC 9027	*C. albicans* ATCC 10231, *Aspergillus brasiliensis* ATCC 16404	0.62–2.5 μL/mL ^▲^	[102]
*Lippia sidoides*	thymol, carvacrol	*S. mutans* ss-980, *S. mitis* (clinical isolates), *S. sanguis* (clinical isolates), *S. salivarius* (clinical isolates)	-	*C. albicans* ATCC 10239	2.5–10 mg/mL ^●^	[50]
	thymol, p-cymene, ether ethyl carvacrol	*Enterococcus faecalis* ATCC 4083	-	-	2.5% ^♠^	[53]
	Thymol	*S. sanguis* ATCC 10556, *S. mitis* ATCC 903	*F. nucleatum* ATCC 25586, *Porphyromonas gingivalis* ATCC 33277	*C. albicans* CBS 562	0.125–0.250 mg/mL ^▲^	[93]
	No data	*S. aureus* (13 clinical isolates)	*E. coli* (13 clinical isolates)	-	3–13 μL/mL ^●^	[103]
	No data	*S. aureus* ATCC 25923, *S. aureus* (isolated from milk)	*E. coli* (isolated from milk)	-	80–320 μL/mL ^●^	[104]
	thymol, p-cymene, ether-methyl carvacrol	-	-	*C. albicans* (8 biological isolated), *C. tropicalis* (6 biological isolated), *C. krusei* (2 biological isolated)	64–256 μg/mL ^▲^	[105]
*Lippia turbinata*	carvone, limonene, β-caryophyllene, 1,8-cineole	*S. aureus* ATCC 25923, *S. aureus* ATCC 29213, *S. epidermidis* ATCC 12228, *E. faecalis* ATCC 29212, *S. aureus* (i.c.)	*E. coli* ATCC 35218, *P. aeruginosa* ATCC 27853, *K. pneumoniae*, *E. cloacae*	-	3.64–29.13 μL/mL ^▲^	[82]
*Phyla dulcis*	β-caryophyllene, δ-cadinene	*S. aureus*	*E. coli*, *S. Tiphymurium*	-	No active	[99]
*Stachytarpheta cayennensis*	No data	-	*E. coli* (13 serotypes clinical strains)	-	≥900 μg/mL ^▲^	[81]
	No data	-	-	*C. albicans* ATCC 10231	0.25 mg/mL ^▲^	[84]

References: minimal inhibitory concentration (MIC) values determined by: ♣ Disk Diffusion Method, * Drop Agar Diffusion Method, ● Broth Dilution Method, ▲ Broth Microdilution Method, ◊ Well Diffusion Method. ♠ MIC values for inhibition of biofilm formation. ♦ The authors no reports MIC values.

**Table 3:** Synergistic interactions between EOs of plants belonging to the Verbenacea family and antibiotics used for human infectious diseases.

Botanical Name	Antibiotic	Strains		Reference
		Gram Positive	Gram Negative	
*Lantana caatingensis*	Neomycin, amikacin, gentamicin	*S. aureus* 358 (multiresistant clinical strain)	*E. coli* 27 (multiresistant clinical strain)	[16]
*L. camara*	Amikacin, gentamicin	*S. aureus*	*P. aeruginosa*	[24]
	Kanamycin, amikacin, gentamicin	*Proteus vulgaris* ATCC 13315, *S. aureus* ATCC 10390	No data	[26]
*L. montevidensis*	Gentamicin, amikacin	*S. aureus* ATCC 12692	*P. aeruginosa* ATCC 15442	[25]
	Neomycin, kanamycin, amikacin, gentamicin	*Proteus vulgaris* ATCC 13315, *S. aureus* ATCC 10390	No data	[26]
	Amikacin, neomycin, gentamicin, kanamycin	*S. aureus* Sa358 (clinical strain)	*E. coli* Ec27 (clinical strain)	[27]
*Lippia alba*	Erythromycin	*S. aureus* ATCC 12692, ATCC 25923, and ATCC 6538	No data	[37]
*L. gracilis*	Kanamycin, gentamicin, tobramycin, amikacin, gentamicin	*S. aureus* ATCC 12692	*E. coli* 27 (clinical strain) *E. coli* ATCC 10536	[40]
*L. origanoides*	Amikacin, neomycin	*S. aureus* SA10 (MRSA clinical isolated), *S. aureus* ATCC 25923	No data	[46]
*L. sidoides*	Gentamicin, amikacin, neomycin	*S. aureus* ATCC 12624	*P. aeruginosa* ATCC 15442	[52]
	Gentamicin, neomycin, penicillin G, ceftriaxone	*S. aureus* ATCC 12624, *S. mutans* ATCC 446, *E. faecalis* ATCC 4083, *E. coli* ATCC 25922, *E. cloacae* ATCC 23355, *K. pneumoniae* ATCC 1003, *Providencia rettigeri*, ATCC 29944	*P. aeruginosa* ATCC 15442	[87]
